# 

**Published:** 1905-11-15

**Authors:** 


Vol. LIX. NOVEMBER 15, 1905. No. 11.

### THE DENTAL REGISTER

EDITED BY

NELVILLE S. HOFF, D.D.S.,

ANN ARBOR, MICH.

Published Monthly by

SAMUEL A. CROCKER & CO.,

Ohio Dental and Surgical Depot,

35 to 39 W. 5th St., Cincinnati, O.

Entered at the Postoffice at Cincinnati O. as second-class mail matter.

